# Bacterial diversity in the surface sediments of the hypoxic zone near the Changjiang Estuary and in the East China Sea

**DOI:** 10.1002/mbo3.330

**Published:** 2016-01-27

**Authors:** Qi Ye, Ying Wu, Zhuoyi Zhu, Xiaona Wang, Zhongqiao Li, Jing Zhang

**Affiliations:** ^1^ State Key Laboratory of Estuarine and Coastal ResearchEast China Normal UniversityShanghai200062China

**Keywords:** Bacteria, Changjiang Estuary, hypoxia, Miseq Illumina sequencing, sediment

## Abstract

Changjiang (Yangtze River) Estuary has experienced severe hypoxia since the 1950s. In order to investigate potential ecological functions of key microorganisms in relation to hypoxia, we performed 16S rRNA‐based Illumina Miseq sequencing to explore the bacterial diversity in the surface sediments of the hypoxic zone near the Changjiang Estuary and in the East China Sea (ECS). The results showed that numerous *Proteobacteria*‐affiliated sequences in the sediments of the inner continental shelf were related to both sulfate‐reducing and sulfur‐oxidizing bacteria, suggesting an active sulfur cycle in this area. Many sequences retrieved from the hypoxic zone were also related to *Planctomycetes* from two marine upwelling systems, which may be involved in the initial breakdown of sulfated heteropolysaccharides. *Bacteroidetes*, which is expected to degrade high‐molecular‐weight organic matter, was abundant in all the studied stations except for station A8, which was the deepest and possessed the largest grain size. In addition, dissolved organic carbon, water depth, percentage ratio of clay to silt, salinity, and sedimentary grain size were environmental effectors that shaped the sedimentary microbial community structure. Our results showed that putative *Gammaproteobacteria*‐affiliated sulfur‐oxidizing bacteria may not only detoxify hydrogen sulfide produced by sulfate‐reducing prokaryotes, but also serve as the primary producers in the marine sediments. Specific groups of aerobic *Bacteroidetes* and *Planctomycetes* participated in degrading organic matter, which might contribute to the oxygen depletion in the hypoxic zones.

## Introduction

Coastal hypoxia, dissolved oxygen (DO) concentration less than 2 mg/L or 62.5 *μ*M in the bottom waters (Vaquer‐Sunyer and Duarte 2008), has been extensively documented in various marine systems such as riverine‐influenced estuaries, highly productive upwelling regions, seasonally stratified fjords, and semienclosed basins (Zhang et al. 2010). Hypoxia that develops in coastal areas off large rivers, such as Changjiang (Wei et al. 2007) and Mississippi rivers (Scavia et al. 2003), are usually linked to combined effects of anthropogenic and physical processes. The river‐dominated estuaries and coastal zones with hypoxia could have direct or indirect effects on the food‐web structure (Baird et al. 2004), nutrient biogeochemical cycles (Howarth et al. 2011), and benthic habitats (Middelburg and Levin 2009). In the coastal area, eutrophication first leads to increased organic matter deposition into the sediments, subsequent promotion of microbial growth and respiration, and finally a greater oxygen demand (Diaz and Rosenberg 2008). Both aerobic and anaerobic sedimentary prokaryotes are key players in the degradation of organic matters. For examples, members of the *Bacteroidetes* are well known for degrading high‐molecular‐weight organic matter (Kirchman 2002). *Planctomycetes* may be involved in the initial breakdown of complex carbohydrates in marine sediments (Glöckner et al. 2003). Bacterial sulfate reduction accounts for up to 50% of the organic matter respiration in coastal marine sediments (Jørgensen 1982).

The Changjiang Estuary and adjacent East China Sea (ECS) are an exceptionally complex and dynamic aquatic ecosystem, due to the mixing of fresh and brine waters and significant recycling of nutrients and organic matters (Zhang et al. 2007a). Changjiang has also suffered serious environmental problems, including eutrophication (Zhang et al. 2007b), harmful algal blooms (Zhou et al. 2008), and oxygen depletion in the near‐bottom waters (Chen et al. 2007). Since hypoxia off Changjiang Estuary was first reported in 1959 (Gu 1980), a number of studies have documented summer hypoxia adjacent to the Changjiang Estuary (Li et al. 2002; Wei et al. 2007; Zhu et al. 2011). Previous studies showed that development of seasonal hypoxia off the Changjiang Estuary resulted from the combined physical (i.e., strong thermal stratification), chemical (e.g., high nutrient loading/organic matter input from the rivers), and biological (i.e., high biological oxygen demand for organic matter mineralization) processes (Rabouille et al. 2008). Because sedimentary oxygen consumption by heterotrophs might trigger hypoxia in bottom waters, the microbial diversity in sediments in the hypoxic area near the Changjiang Estuary were investigated. The goal was to learn more about the potential ecological roles of the key microorganisms.

There has been a growing research effort on microbial ecology in Changjiang Estuary and in the ECS (Jiao et al. 2007; Feng et al. 2009; Liu et al. 2012; Dong et al. 2014). Based on 16S rRNA gene clone libraries, differences in the bacterial communities were found between water and sediments, with seasonal and spatial variation in the seawater and sediments also being observed (Feng et al. 2009). Temporal distribution of the bacterial community of seawater in the Changjiang Estuary hypoxia area and its adjacent area in the ECS were examined by denaturing gradient gel electrophoresis analysis (Liu et al. 2012). In addition, both estimated richness and diversity indices were found higher in the sediment samples than the seawater samples in the ECS by the 454 pyrosequencing method (Dong et al. 2014). However, little is known about microbial community and the effects of environmental factors on microbial community distribution in the surface sediments off Changjiang Estuary during hypoxia. During a cruise in August 2013, we observed three hypoxic subzones, with a combined surface area of up to 11,000 km^2^, near the Changjiang Estuary. In this study, 16S rRNA gene‐based Miseq Illumina sequencing method was used to investigate the microbial community compositions, diversity, and distribution in surface sediments underlying hypoxic condition and in the ECS.

## Experimental Procedures

### Site descriptions, sample collections, and in situ measurement

Changjiang is the largest river in Asia and produces the fourth largest runoff in the world. The summer hypoxia in the waters adjacent to Changjiang Estuary has become more common in the past several decades. The center of the hypoxic zones has been around 123°E, 31°N. In recent years, the location has been moving northward (Zhu et al. 2011).

In August 2013, a series of sediments (upper 3 cm) from Changjiang Estuary and in the ECS were collected by box corer on the R/V Dongfanghong #2. A total of 12 stations were selected for microbial community analysis. These sediment samples were stored at −20°C onboard, transported on ice, and stored at −80°C in the laboratory until DNA extraction. In situ physical profiles (i.e., temperature and salinity with depth) were obtained by CTD (Sea‐Bird^®^ 11 plus, Sea‐Bird Electronics, Bellevue, WS, USA). DO was measured by a DO meter, and the presented data were further calibrated by the Winkler method. Dissolved oxygen carbon (DOC) samples were filtered via clean Nylon filter (pore size: 0.45 *μ*m) immediately after collection and kept at −20°C until analysis. In the laboratory, DOC samples were measured with a TOC analyzer (Shimadzu^®^ TOC‐L_CPH_, Japan). Grain sizes of sediment samples were determined using a laser particle size analyzer (LS‐100Q, Beckman Coulter Corporation, Fullerton, CA, USA) as described previously (Luo et al. 2012). The strength of stratification (Δ*σ*) was calculated as described previously (Zhu et al. 2011).

### DNA extraction and PCR

Total DNA was extracted from ~0.5 to 1 g sediment samples using a MoBio PowerSoil^®^ DNA Isolation Kit (MOBIO Laboratories, Carlsbad, CA, USA) according to the manufacturer's instruction. DNA from three independent extractions were combined and their concentration and purity were measured spectrophotometrically with NanoDrop ND2000 (Thermo Fisher Scientific, Wilmington, DE, USA).

In order to decrease PCR bias, minimum numbers of PCR cycles were chosen and three independent PCR mixtures were pooled for each sample. Bacterial V4–V5 hypervariable regions of 16S rRNA genes were amplified using the specific barcoded universal primer pairs 515F (5'‐GTGCCAGCMGCCGCGG‐3') and 907R (5'‐CCGTCAATTCMTTTRAGTTT‐3'). PCR amplification were performed in an ABI GeneAmp^®^ 9700 (Applied Biosystems, Foster City, CA, USA) using an initial denaturing step of 95°C for 2 min, followed by 25 cycles of 95°C for 30 sec, 55°C for 30 sec, and 72°C for 30 sec, and then an elongation step of 72°C for 5 min.

### Miseq Illumina sequencing

PCR products were purified using the AxyPreDNA gel extraction kit (Axygen Biosciences, Union City, CA, USA) following the manufacturer's protocol. PCR products were quantified by QuantiFluorTM‐ST (Promega, Madison, WI, USA). Reaction mixtures were pooled in equimolar ratios and paired‐end reads were generated on an Ilumina Miseq PE250 (Majorbio Bio‐Pharm Technology Co., Ltd., Shanghai, China).

### Sequence data process

Raw fastq files were demultiplexed, quality‐filtered using QIIME (version 1.17) (Caporaso et al. 2010) with the criteria as described previously (Li et al. 2014).

### Operational taxonomic units cluster and taxonomic assignment

Operational taxonomic units (OTUs) were clustered with 97% similarity cutoff using UPARSE (version 7.1, http://drive5.com/uparse/). Chimeric sequences were identified and removed using UCHIME (version 4.2.40, http://drive5.com/usearch/manual/uchime_algo.html). An “OTU table,” showing the number of reads from each sample that was assigned to each OTU, was created by using the usearch_global command. Representative 16S rRNA gene sequences of each OTU were analyzed by RDP Classifier (http://rdp.cme.msu.edu/) against the Silva (SSU115) 16S rRNA database using confidence threshold of 70%.

### Phylogenetic analyses

The sequences of representative OTUs and reference sequences from the database were aligned using Clustal W, and phylogenetic trees were generated in MEGA6 using the neighbor‐joining method with a bootstrap test of 1000 replicates and maximum composite likelihood model (Tamura et al. 2013).

### Statistical analyses

Alpha diversity metrics were measured using the Mothur Program (Schloss et al. 2009). Coverage was calculated using the equation: [1 − (*n*_1_ / *N*)] × 100, where *n*_1_ is the number of single‐occurrence reads within an OTU and *N* is the total number of reads in the data set.

R program was used to plot Heatmap (R Development Core Team, 2013) R Development Core Team. 2013. R: A language and environment for statistical computing. 2013. Available at: http://www.r-project.org/. Bray–Curtis dissimilarities were calculated based on class/sample abundance matrix using function vegdist of the vegan package in R (Oksanen et al. 2007). Hierarchical clustering of the samples was performed with the complete method using the function hclust of stats package in R (R Development Core Team, 2013).

A multivariate statistical analysis was performed using the vegan package in R. Station F3 was excluded from this analysis due to lack of the information. The abundance of all observed OTUs was used in this analysis. The detrended correspondence analysis was done for all tested samples. The longest gradient length was larger than 3, indicating that all tested samples were suitable for the canonical correspondence analysis (CCA). Seven measured environmental variables were used for CCA analysis, and the relationship between individual environmental variables and microbial communities was examined by a correlation test. Pearson's correlation coefficient was calculated using SPSS (SPSS Inc, Chicago, IL, USA) software. The corresponding heatmap was plotted using the gplots package in R (Warnes et al., 2012).

### Nucleotide sequence accession numbers

Paired‐end Illumina sequence data from this study were submitted to the NCBI Sequence Read Archive (SRA) under accession number SRP055504.

## Results and Discussion

### Sediment types and environmental characteristics

**Figure 1:**
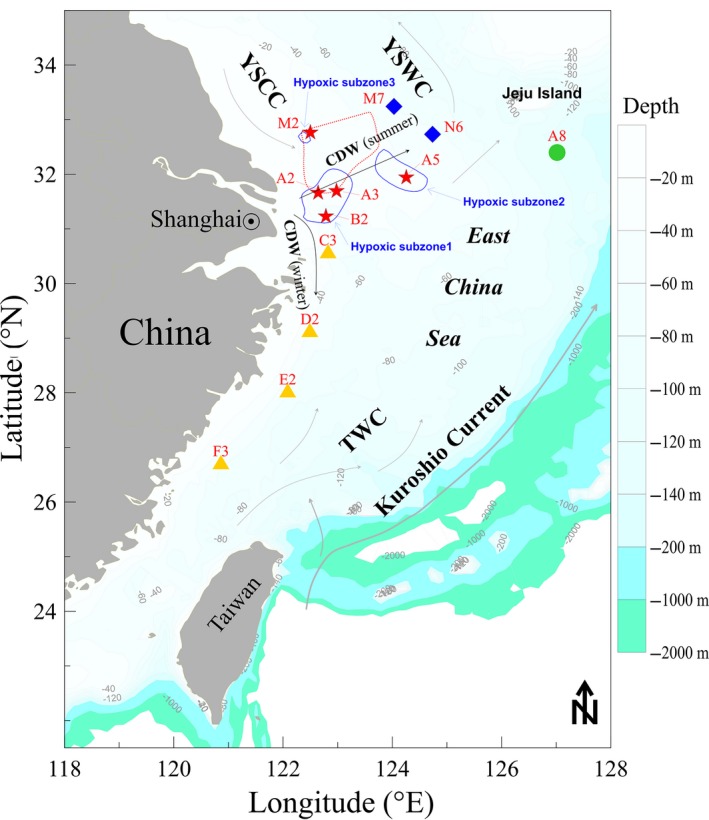
Map showing the sampling stations is classified according to the characteristics of the locations and physicochemical parameters: Group O (**A2**, **A3**, **A5**, **B2**, and **M2**), Group J (**A8**), Group T (**M7** and **N6**), and Group S (**C3**, **D2**, **E3**, and **F3**). The location of the previously reported hypoxic area is indicated by a red dashed line. Three hypoxic subzones were detected in this study indicated by blue solid lines. CDW, Changjiang dilute water; YSCC, Yellow Sea cold current; YSWC, Yellow Sea warm current; TWC, Taiwan warm current.

Sediment types and environmental parameters of 12 studied samples were summarized in Table 1. During the cruise August 2013, three hypoxic subzones were observed just north of the exit of the Changjiang Estuary into the ECS (Fig. 1). Parts of these subzones were in regions where hypoxia had never been detected before (Fig. 1).

**Table 1:** Environmental physicochemical parameters of bottom water at the stations in the hypoxic areas of the Changjiang Estuary and adjacent East China Sea

Station	Depth (m)	Mean grain size (*μ*m)	Clay (%)	Silt (%)	Sand (%)	Δ*σ* (kg/m^3^)	DO (mg/L)	Temperature (°C)	Salinity (PSU)	DOC (*μ*mol/L)
A2	34.3	159.1	8.48	20.92	70.6	10.89	1.79	21.02	33.42	48.92
A3	37.4	197.4	8.42	17.08	74.5	5.11	1.55	21.68	33.16	72.76
A5	41.1	104.8	18.5	40.8	40.7	3.05	1.56	21.29	31.84	81.85
B2	47.3	109.4	21.3	38.4	40.3	6.95	1.80	19.24	34.34	55.32
M2	26	116.9	9.89	26.71	63.4	0.46	2.49	23.99	31.42	84.57
A8	118	319.2	11.2	16.7	72.1	3.40	5.16	15.42	34.52	51.54
M7	66	74.71	23.3	36.6	40.1	5.74	6.30	10.35	32.93	78.97
N6	63.4	118.7	22	29.5	48.5	6.76	5.48	12.18	32.43	88.30
C3	38	18.67	27.5	67.3	5.2	4.26	3.6	19.38	34.37	56.37
D2	46	13.66	34.1	63.2	2.7	3.81	3.3	18.35	34.38	53.57
E2	58	13.24	35.8	61.6	2.6	3.60	5.2	17.68	34.37	51.30
F3	55	15.52	33.7	62.7	3.6	1.55	ND	24.19	33.92	ND

Δ*σ* (strength of stratification), the difference between surface and bottom water density; DO, dissolved oxygen; DOC, dissolved organic carbon; ND, not determined.

Four sediment samples (C3, D2, E2, and F3) located south of the Changjiang Estuary were the most fine grained Station A8, with the deepest sampling depth of 118 m, had the largest mean grain size (318 *μ*m) (Table 1). Strength of stratification (Δ*σ*) is defined as the difference between surface and bottom water density. We found strong stratification in hypoxic subzone 1, and stations M7 and N6. Interestingly, station M2 has the lowest Δ*σ* (0.46 kg m^−3^), which indicated that the water column was mixed vertically (Table 1).

The temperature and salinity of the near‐bottom waters ranged from 10.35°C to 24.19°C and 31.42 PSU (practical salinity units) to 34.52 PSU, respectively. The two stations with the lowest temperatures were M7 (10.35°C) and N6 (12.18°C) (Table 1). DOC of the near‐bottom waters ranged from 48.92 *μ*mol/L at station A2 to 88.30 *μ*mol/L at station N6 (Table 1).

Based on the sampling locations and physicochemical parameters (Table 1, Fig. 1), 12 samples were separated into four groups: Group O included five samples collected from oxygen depletion zones (A2, A3, A5, B2, and M2), where DO concentrations of the near‐bottom waters were below 3 mg/L. Diatom blooms were also observed at the stations in Group O, especially in stations A2 and A3, during the sampling time. Group T included stations M7 and N6, which had low temperatures. Group S included four stations south of off the Changjiang Estuary (C3, D2, E2, and F3) with fine grain sizes. Group J included only station A8 close to Jeju Island with the deepest sampling depth and the largest mean grain size.

### Bacterial diversity and cluster analysis

**Figure 2:**
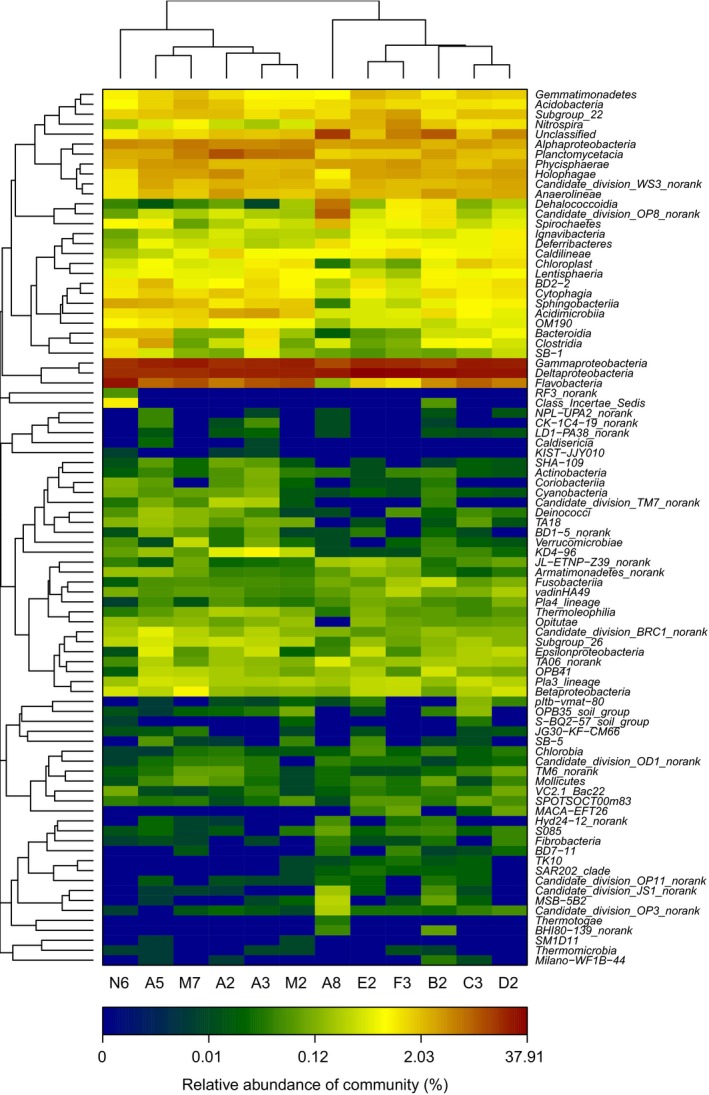
Heatmap showing the relative abundance and distribution of class‐based operational taxonomic unit (OTU) Illumina reads. The color code indicates relative abundance, ranging from blue (low abundance) to black to brown (high abundance). Twelve samples can be organized into two groups: one group was mainly composed of the Group O (A2, A3, A5, and M2) and Group T (M7 and N6), and the other group consisted of the Group S (C3, D2, E2, and F3) and Group J (A8). Station B2 was more similar to stations in Group S and clustered separately from the other stations from Group O.

We investigated the bacterial diversity of the 12 samples. A number of bacterial diversity indices for the 16S rRNA Illumina reads are shown in Table 2. Both community richness indices Chao1 and abundance‐based coverage estimator were lowest in the station A8, followed by stations E2 and N6, but highest in station B2. Good's coverage was 94.5–98.3% for all samples. Station A8 is located in the outer continental shelves of ECS, minimally influenced by terrestrial input, whereas the stations in the areas near the Changjiang Estuary receive a large amount riverine materials (Wu et al. 2013), which may support more diverse bacterial groups. Heatmap analysis at the bacterial class level showed that 12 samples can be organized into two groups: one group was mainly composed of the Groups O and T, and the other group consisted of the Groups S and J. Station B2 was more similar to stations in Group S, and did not cluster with the other stations from Group O. Neither station N6 nor A8 clustered with the other stations, suggesting that they possessed distinctive bacterial communities (Fig. 2). Our results showed the distinctive bacterial distributions among the four sampling groups, which may have been due to environmental selections.

**Table 2:** Diversity and richness estimators for Illumina libraries

Station	Number of sequences	Observed OTU	Chao	ACE	Shannon index	Coverage(%)
A2	24,853	1730	2047	2048	6.11	98.3
A3	16,479	1567	1936	1949	5.98	97.3
A5	25,357	1843	2218	2206	6.12	98.2
B2	18,713	1816	2279	2287	6.18	97.2
M2	16,918	1710	2139	2197	6.07	96.9
A8	13,384	1268	1562	1544	5.75	97.5
M7	18,616	1577	1911	1929	6	97.7
N6	21,445	1455	1852	1852	5.49	98
C3	13,582	1500	1992	2001	5.96	96.2
D2	9207	1335	1870	1904	6.04	94.5
E2	12,061	1356	1783	1810	5.88	96.2
F3	11,988	1423	1877	1896	5.96	96

OTU, operational taxonomic units; Chao 1 species richness; ACE, abundance‐based coverage estimator.

### Bacterial distributions

**Figure 3:**
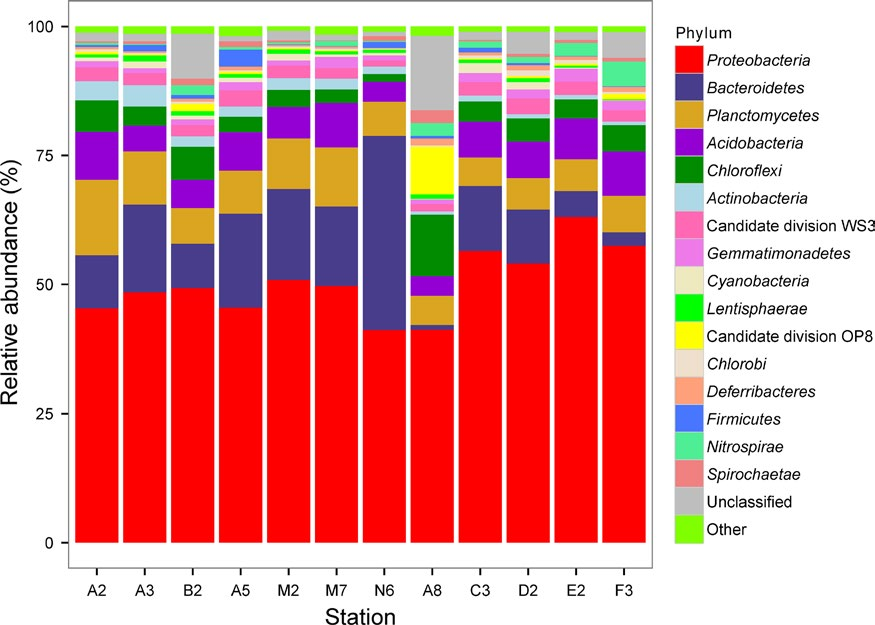
Distributions of phylum‐level taxa. Bars represented the relative abundance of Illumina sequences representative of each phylum. Other phyla that are not named contain less than 1% of the reads.

We obtained a total of 202,603 high‐quality bacterial V4–V5 Illumina sequences, with an average read length of 396 bp from 12 sampling stations. When grouped at the 97% similarity level, there were 2982 OTUs in the complete data set, with an average of 1548 OTUs per station. Of these, 251 OTUs were found in all samples. *Proteobacteria* was the dominant phylum in all samples, ranging from 41.3 to 63.5% of the sequences. *Bacteroidetes* and *Planctomycetes* were another two abundant phyla at the Groups O and T stations. Notably, station N6 contained 37% *Bacteroidetes*. At the stations in Group S, *Bacteroidetes* constituted ~11% of the bacterial sequences at the C3 and D2 stations, and their relative abundance then declined southward (~2.6% in station F3). In addition, *Bacteroidetes* was almost absent from station A8 (<1%). In the Group O stations, *Planctomycetes* was the second most abundant phylum in station A2 (~14.6%), and ranked as the third most abundant phylum in the rest of the stations (6.8–10.3%). Candidate division OP8 accounted for 9.2% of sequences in station A8, but was seldom detected at the other stations. Station A8 also contained the highest number of unclassified sequences among all samples (~14.4%) (Fig. 3, Table S1).

### Putative sulfate‐reducing and sulfur‐oxidizing bacteria

**Figure 4:**
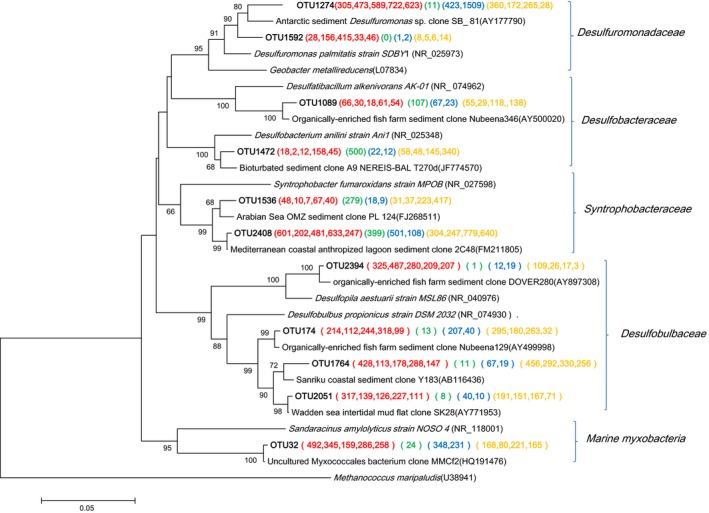
Neighbor‐joining phylogenetic tree of *Deltaproteobacteria* affiliated 16S rRNA sequences of the representative operational taxonomic units (OTUs) and their closest environmental sample entries in the NCBI GenBank. These OTUs were represented by more than 1% of the reads from a single sample and/or from multiple samples. The OTUs obtained in this study are shown in bold type. The scale bar represents the estimated number of nucleotide changes per sequence position. The symbols at the nodes show the bootstrap values (only those >50% are indicated) obtained after 1000 resamplings. The numbers in parentheses indicate the number of reads in each station in the following order (Group O, Group J, Group T, and Group S) and color (**A2**, **A3**, **A5**, **B2**, and **M2**), (**A8**), (**M7** and **N6**), and (**C3**, **D2**, **E2**, and **F3**), respectively. *Methanococcus maripaludis* (U38941) was used as the out‐group.

Eleven deltaproteobacterial‐related OTUs were represented by more than 1% of the reads from either a single sample or from multiple samples. Ten of these OTUs were likely to be sulfate‐reducing bacteria (SRB) based on the phenotypes of their close relatives (Fig. 4). These SRB‐related OTUs were affiliated with four families: *Desulfuromonadaceae, Desulfobacteraceae, Desulfobulbaceae,* and *Syntrophobacteraceae,* which were similar in the shallow sediments underlying hypoxic waters in the northern Gulf of Mexico on the Louisiana shelf (Reese et al. 2013).

The composition of SRB among the four groups of sampling stations differed (Fig. 4). For example, sequences affiliated with families *Desulfobulbaceae* and *Desulfuromonadaceae* were seldom recovered from station A8. The sequence OTU2394 was the most frequently obtained from Group O, and this OTU was closely related to *Desulfopila aestuarii* MSL86 (~96% similarity) and an environmental clone retrieved from organically enriched fish farm sediment (Bissett et al. 2006). *Desulfopila aestuarii* MSL86, isolated from estuarine sediments in Japan, could incompletely oxidize a variety of substrates to acetate (Suzuki et al. 2007a). Three OTUs in Desulfobulbaceae were frequently detected in all stations, but A8. Their closest cultured relative was *Desulfobulbus propionicus* (92–94% sequence identity with OTU174, 1764, 2051). Members of the genus *Desulfobulbus* usually incompletely oxidize organic electron donors to acetate (Suzuki et al. 2007b). OTU1274 in the *Desulfuromonadaceae* family was frequently found in Groups O, T, and S stations. It had ~96% similarity to *Desulfuromonas* sp. clone SB2_ 81 that had been detected in Antarctica coastal sediment with high productivity (Purdy et al. 2003). It seems that acetate produced by SRB within *Desulfobulbaceae* may be utilized by sulfur‐reducing *Desulfuromonas* in the sediments of inner continental shelf. OTU1472 included 500 sequences from station A8 that displayed ~97% similarity with *Desulfobacterium anilini*. OTU2408 within *Syntrophobacteraceae* was common at all stations. It had 99% similarity with an environmental clone found in heavy metal and hydrocarbon contaminated sediment in Bizerte lagoon from the Southern Mediterranean (Said et al. 2010). Members of the family *Syntrophobacteraceae* could oxidize propionate in syntrophic coculture or as a pure culture coupled to dissimilatory sulfate reduction (Plugge et al. 2011). Our results showed different SRB groups partitioned in different sampling stations, and we speculate that different types of substrates support the growth of specialized groups of SRB.

**Figure 5:**
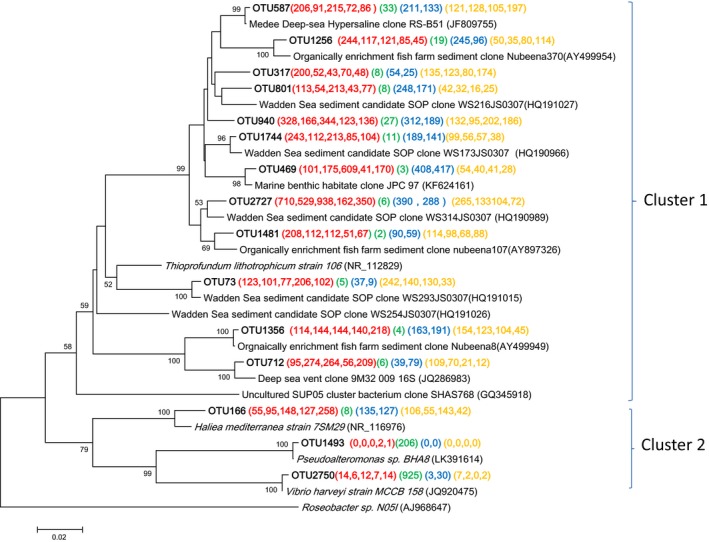
Neighbor‐joining phylogenetic tree of *Gammaproteobacteria* affiliated 16S rRNA sequences of the representative operational taxonomic units (OTUs) and their closest environmental sample entries in the NCBI GenBank. These OTUs were represented by more than 1% of the reads from a single sample and/or from multiple samples. The OTUs obtained in this study are shown in bold type. The scale bar represents the estimated number of nucleotide changes per sequence position. The symbols at the nodes show the bootstrap values (only those >50% are indicated) obtained after 1000 resamplings. The numbers in parentheses indicate the number of reads in each station in the following order (Group O, Group J, Group T, and Group S) and color (**A2**, **A3**, **A5**, **B2**, and **M2**), (**A8**), (**M7** and **N6**), (**C3**, **D2**, **E2**, and **F3**). *Roseobacter* sp. N05I (AJ968647) was used as the out‐group.

Fifteen *Gammaproteobacteria* OTUs possessed high abundances, that is, possessed more than 1% of the reads from either a single sample or from multiple samples. They formed two distinctive clusters (Fig. 5). The six OTUs in Cluster 1 were closely related to likely sulfur‐oxidizing bacterial (SOB) clones from Wadden Sea sediments (Lenk et al. 2011), and the sequences of OTU73, OTU1744, and OTU2727 possessed >98% similarity with these clones. OTU1256, OTU1356, and OTU1481 had 98–99% similarity with clones from fish farm sediments, suggesting that they were adapted sediments enriched in organic matter (Bissett et al. 2006). Sequences of OTUs in Cluster 1 were seldom recovered from station A8. Three OTUs in Cluster 2 were affiliated with cultured *Gammaproteobacteria*. The sequences represented by OTU166 were related to *Hailea mediterranea*, an aerobic marine *Gammaproteobacteria* which could reduce nitrate to nitrite, degrade several polymers, and utilize organic acids and amino acids as carbon and energy sources (Lucena et al. 2010). OTU1493 and 2750 were recovered almost exclusively from station A8. These 16S rRNA sequences were identical to those of *Pseudoalteromonas* sp. and *Vibrio harveyi* species, respectively.

Although SRB in organic‐rich coastal sediments produce a large amount of hydrogen sulfide, most of this toxic metabolite is reoxidized at the sediment–water interface (Jansen et al. 2009). Sediment surfaces under hypoxic conditions are commonly covered with microbial mats formed by SOB, such as *Beggiatoa*,* Thioploca*, and *Thiomargarita*, which are capable of removing most of the sulfide using nitrate as an electron acceptor (Levin et al. 2009). The sulfur‐oxidizing *Epsilonproteobacteria Sulfurimonas* spp. represents the major chemoautotrophs in the pelgic redoxclines of the Baltic and Black Seas (Grote et al. 2008). It has been shown previously that the SRB and *Sulfurimonas* spp. were abundant in the hypoxia treatments during a 28‐days microcosm experiment of surface sediments (Chan et al. 2014). Reese et al. (2013) demonstrated that a majority of the reoxidation of sulfide in the shallow sediments in the hypoxic zone of northern Gulf of Mexico was due to SOB, including members of the families *Ectothiorhodospiraceae, Thiotricaceae*, and *Chromatiaceae*. A recent study on the alphaproteobacterial *Roseobacter* clade (RCB) suggested that they oxidized inorganic and organic sulfur compounds in oxic and suboxic sediment layers, possibly using nitrate as an electron acceptor to overcome oxygen limitation (Lenk et al. 2012). Novel groups of uncultured *Gammaproteobacteria* use sulfur as electron donor coupling to 40–70% of the CO_2_ fixation were reported in a coastal intertidal sediment in the Wadden Sea (Lenk et al. 2011). In our study, very few retrieved sequences were related to *Epsilonproteobacteria*, RCB, and other SOB commonly detected under hypoxic conditions. Instead, we detected six Wadden Sea Candidate SOB‐affiliated OTUs. They represented 3.7–7.2% of the sequences within samples from Groups O, T, and S stations. In oxygen‐free sediments, the SOB may use alternative electron acceptors such as conductive solids (Nielsen et al. 2010), but it remains unknown how these uncultured candidate *Gammaproteobacteria* compete with metal‐catalyzed sulfide oxidation. The fixed carbon produced by the autotrophic SOB may feed both heterotrophic bacteria and sediment fauna, playing important roles in the benthic food web of coastal marine sediments (Boschker et al. 2014). The SOB not only prevents toxic hydrogen sulfide from diffusing into the bottom water, but they contribute primary production to the marine sediments. For this reason, the ecological functions of SOB in the sediments near the Changjiang Estuary deserve further attention.

### Diversity of *Planctomycetes*

**Figure 6:**
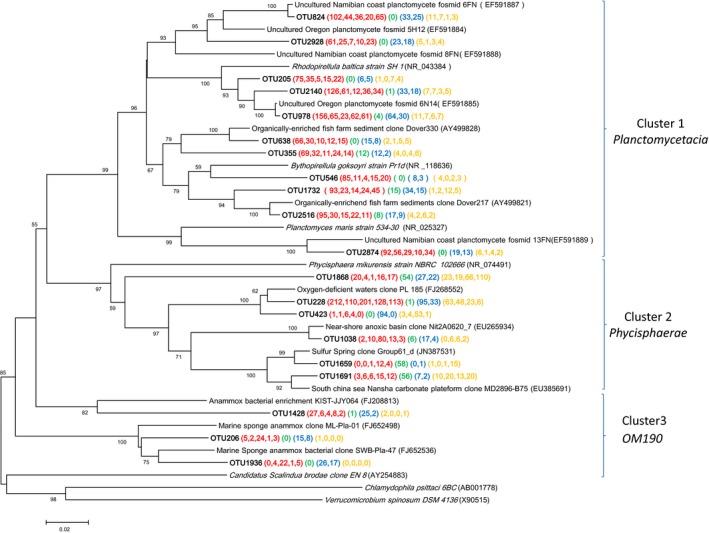
Neighbor‐joining phylogenetic tree of *Planctomycetes* affiliated 16S rRNA sequences of the representative operational taxonomic units (OTUs) and their closest environmental sample entries in the NCBI GenBank. OTUs contained more than 50 sequences within *Planctomycetacia* or *Phycisphaerae* and 20 sequences within OM190 from a single sample and/or from multiple samples. The OTUs obtained in this study are shown in bold type. The scale bar represents the estimated number of nucleotide changes per sequence position. The symbols at the nodes show the bootstrap values (only those >50% are indicated) obtained after 1000 resamplings. The numbers in parentheses indicate the number of reads in each station in the following order (Group O, Group J, Group T, and Group S) and color (**A2**, **A3**, **A5**, **B2**, and **M2**), (**A8**), (**M7** and **N6**), and (**C3**, **D2**, **E2**, and **F3**). *Chlamydophila psittaci* 6BC (AB01778) and *Verrucomicrobium spinosum* DSM4136 (X90515) were used as out‐groups.

In station A2, approximately 14.6% sequences fell into the family *Planctomycetaceae*. Sequences from this family were also common at stations A3 (10.3%) and M2 (9.8%), both of which were depleted for O_2_ (Fig. S1). Considering low abundance of *Planctomycetes*‐related OTUs, OTUs contained more than 50 sequences within *Planctomycetacia* or *Phycisphaerae* and 20 sequences within *OM190* in single sample and/or in multiple samples were selected for phylogenetic analysis, and these OTUs formed three clusters (Fig. 6). Cluster 1 comprised OTU824, which exhibited 99% similarity with fosmid 6FN, and OTU2874, which had 96% similarity with fosmid 13FN. These fosmids were retrieved from the OMZ of the Benguela upwelling system at the Namibian coast (Woebken et al. 2007). The cultured relative of OUT2874 was *Planctomyces maris* (86% identity). Three OTUs 205, 978, and 2140 showed 97–99% similarity with fosmid 6N14, which was retrieved from 200‐m depth off the coast of Oregon (Woebken et al. 2007). They were also related to *Rhodopirellula baltica* strain SH 1 (~95% identity). Cluster 2 included OTUs related to *Phycisphaera mikurensis*. More than 100 sequences from the O_2_‐depleted stations were grouped into OTU228, which possessed 97% identity with an environmental clone from the organic‐rich sediment underlying O_2_‐deficient waters of the eastern Arabian Sea (Divya et al. 2011). A few sequences in three OTUs within OM190 were distantly related to anammox‐like environmental sequences in Cluster 3 (Fig. 6).

Members of the aerobic planctomycetes are ubiquitous in aquatic and terrestrial environments and now thought to be significant contributors to carbon recycling (Lage and Bondoso 2014). Several lines of evidence indicate that aerobic planctomycetes increase in abundance in response to algal blooms (Morris et al. 2006; Pizzetti et al. 2011a,b). *Pirellula* and OM43 were identified among the dominant lineages in an Oregon coast diatom bloom (Morris et al. 2006). Pizzetti et al. (2011a) found that planctomycete abundance correlated positively with chl a and DOC, which indicated fresh substrates provided by summer algal bloom may support the growth of the *Planctomycetes*. Temporal variability of coastal *Planctomycetes* clades at Kabeltonne Station from North Sea showed strong links between specific clades of *Planctomycetes* and particular groups of algae (Pizzetti et al. 2011b). Furthermore, genomic and metagenomic analysis revealed the specialization of marine planctomycetes for degrading algal polymers (Glöckner et al. 2003; Woebken et al. 2007). The marine strain *Rhodopirellula baltica* SH 1^T^ encodes high numbers of sulfatases (Glöckner et al. 2003). Fosmids 6FN and 5H12 from two marine upwelling systems also contain sulfatase. These are known to be involved in efficient degradation of complex sulfated heteropolysaccharides.

Strong stratification was detected based on the Δ*σ* measurement (>5 kg m^−3^) in hypoxic subzone 1. Δ*σ* is clearly positively related to the bottom apparent oxygen utilization (AOU) until Δ*σ* reaches 5 kg m^−3^. When strong stratification exists (>5 kg m^−3^), the bottom AOU increased slowly or remained stable, and other O_2_ consumption processes such as organic matter decomposition determines the bottom AOU (Zhu et al. 2011). We observed diatom blooms in the hypoxic subzone 1. The diatom species usually sink to the bottom layer and then release many organic materials. The strong stratification in hypoxic subzone 1 prevented the vertical DOC exchanges, trapping part of the dissolved organic materials in the sediments, and providing sufficient substrates for planctomycete growth. Sequences in cluster 1 within *Planctomycetes* (Fig. 6) from hypoxic subzone 1 had high‐level identity with sulfatase‐contained fosmids and marine planctomycetes *Rhodopirellula baltica SH 1*, suggesting their ecological importance in relation to algal blooms. Our results have led to the hypothesis that decomposition of algal‐derived organic matter by specific groups within class *Planctomycetacia* might play a significant role in O_2_ depletion.

### Diversity of *Bacteroidetes*

**Figure 7:**
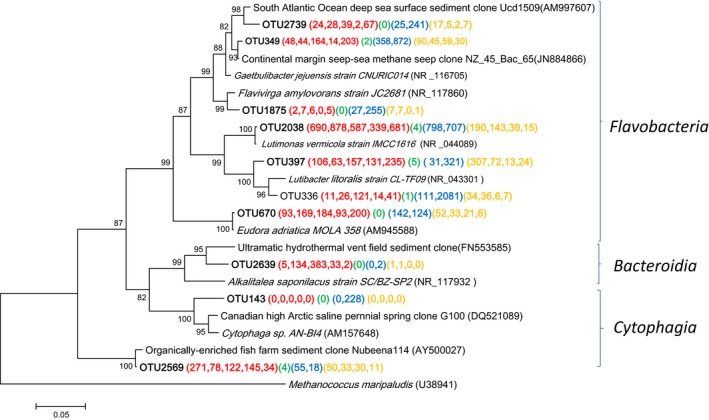
Neighbor‐joining phylogenetic tree of *Bacteroidetes* affiliated 16S rRNA sequences of the representative operational taxonomic units (OTUs) and their closest environmental sample entries in the NCBI GenBank. These OTUs were represented by more than 1% of the reads from a single sample and/or from multiple samples. The OTUs obtained in this study are shown in bold type. The scale bar represents the estimated number of nucleotide changes per sequence position. The symbols at the nodes show the bootstrap values (only those >50% are indicated) obtained after 1000 resamplings. The numbers in parentheses indicate the number of reads in each station in the following order (Group O, Group J, Group T, and Group S) and color (**A2**, **A3**, **A5**, **B2**, and **M2**), (**A8**), (**M7** and **N6**), and (**C3**, **D2**, **E2**, **and** **F3**). *Methanococcus maripaludis* (U38941) was used as the out‐group.

Among phylum *Bacteroidetes*, the relative abundance of sequences within the family *Flavobacteriaceae* varied from 0.15% in station A8 to 26.74% in station N6 (Fig. S2). There were 10 OTUs represented by more than 1% of the reads from either a single sample or from multiple samples (Fig. 7). Three OTUs displayed relative high identities (96–99%) with cultured representatives within the family *Flavobacteriaceae*. OTU2038 exhibited 99.7% similarity with obligately aerobic *Lutimonas vermicola* IMCC1616^T^, and OTU397 also showed ~96% similarity with this strain (Yang et al. 2007). OTU336, which included 9.73% sequences at station N6, had 98.9% similarity with obligately aerobic *Lutibacter litoralis* CL*‐*TF09^T^ isolated from tidal flat sediment in Ganghwa, Korea (Choi and Cho 2006). The phylogenetic affiliation of the environmental sequences to cultured *Flavobacteriaceae*, especially with the genus *Lutimonas* and *Lutibacter*, may indicate that they are dominant *Flavobacteriaceae* in all stations, but A8. Many members of the family *Flavobacteriaceae* have been isolated from marine environments and are essential for the organic matter mineralization in marine ecosystems (Cottrell and Kirchman 2000). In the sediments studied here, the *Flavobacteriaceae* may have participated in degrading high‐molecular‐weight organic matter, making labile compounds available to the other groups of microorganisms. In this fashion, the aerobic *Flavobacteriaceae* may also have contributed to the O_2_ depletion in this area.

### Relationships between microbial community structure and environmental variables

**Figure 8:**
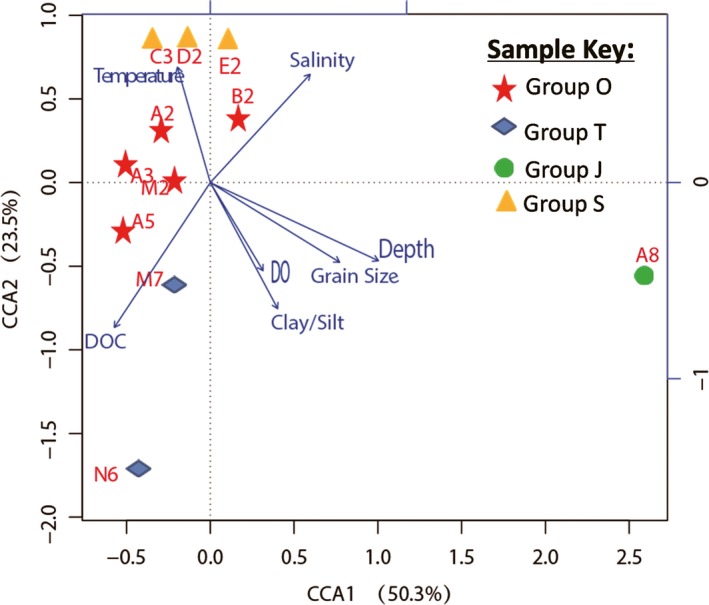
Canonical correspondence analysis (CCA) illustrating the relationship between the operational taxonomic unit (OTU)‐level community structure of the sampling sites and selected environmental variables.

In a CCA of the abundances of taxonomic groups with environmental variables, the first axis showed the highest positive correlation with sampling water depth and mean grain sizes, distinguishing station A8 from the other stations (Fig. 8). Salinity and temperature showed a positive correlation with the second axis, while DOC, ratio of clay to silt, and DO were the parameters with the negative correlations. This second axis was able to discriminate among four sampling groups. Pr shows the significance of correlation test of individual environmental variable and microbial communities. The Pr results revealed that DOC, water depth, ratio of clay to silt, salinity, and mean grain size, but not DO and temperature, were the environmental effectors that played major roles on the distribution of sedimentary microbial communities (Table S2). In our study, we found DOC was one of the potentially significant effectors influencing the sedimentary bacterial community. This may be explained that a large amount of DOC that was produced by phytoplankton near the estuary mouth and different sources of DOC (Wu et al. 2013).

**Figure 9:**
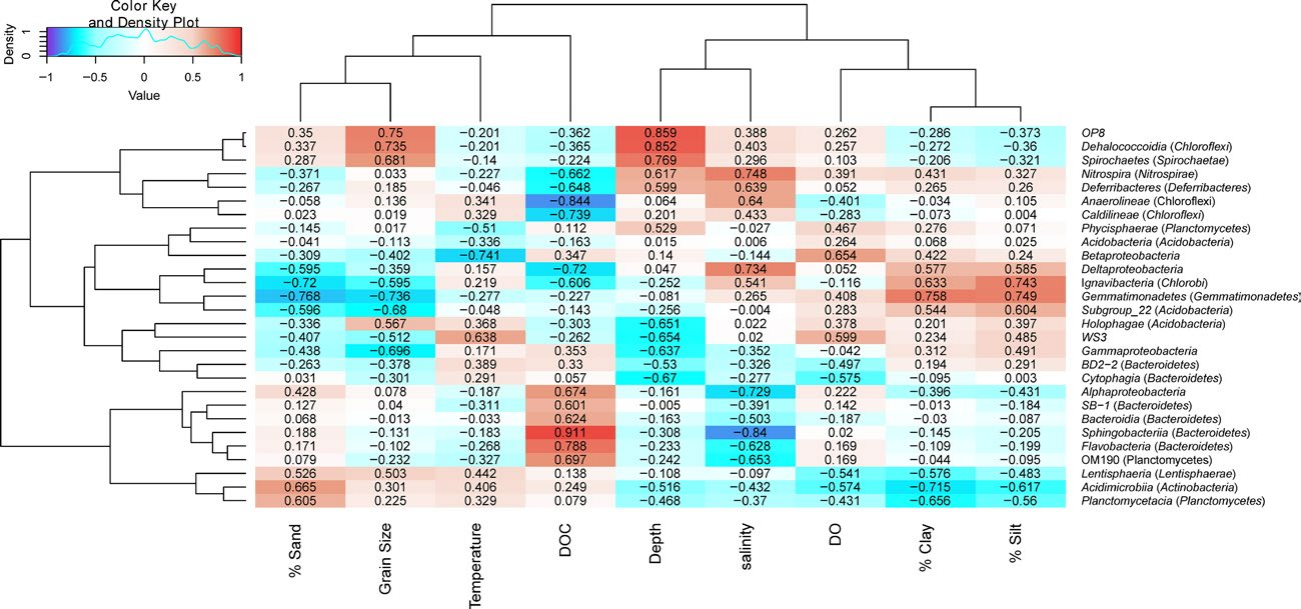
Environmental factors associated with variations in the bacterial community structure at the class level. The phylum for each class is indicated in parentheses. Pearson's correlation coefficients between −1 and 1 are shown in the rectangle, which indicates correlations between class sequence abundance and selected environmental parameters. For example, a firebrick colored rectangle (0.911) between DOC and *Sphingobacteria* indicates a higher number of *Sphingobacteria* sequences with increasing DOC concentrations; a blue rectangle (−0.84) between salinity and *Sphingobacteria* indicates a higher number of sequences with decreasing salinity; the absence of a significant relationship between DO and number of *Sphingobacteria* sequences is indicated by white rectangle (0.02). The color code indicates Pearson's correlation coefficients, ranging from blue (−1) to white (0) to firebrick (1). The density showed the distribution of Pearson's correlation coefficients between −1 and 1. DOC, dissolved organic carbon, DO, dissolved oxygen; % clay, clay percentage; % silt, silt percentage; % sand, sand percentage.

**Figure 10:**
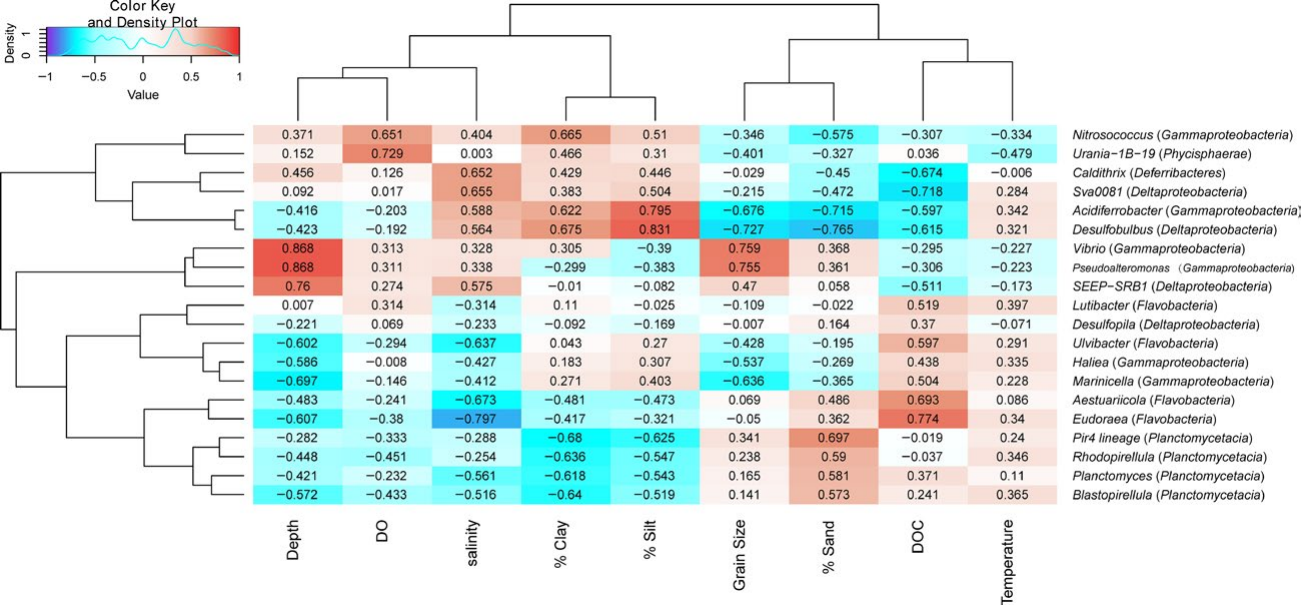
Environmental factors associated with variations in the bacterial community structure at the genus level. The class for each genus is indicated in parentheses. Pearson's correlation coefficients between −1 and 1 are shown in the rectangle, which indicates correlations between genus sequence abundance and selected environmental parameters. See the legend for Figure 9 for additional explanations.

In order to further investigate relationships between bacterial taxonomic groups and environmental conditions, we calculated the Pearson's correlation coefficient between some bacterial taxonomic groups and contextual environmental parameters (Gobet et al., 2012). The selected classes/genera were represented by more than 1% of the reads from at least one station. We found significant Pearson's correlation coefficients between DOC of the near‐bottom waters and *Alphaproteobacteria*, class *OM190* within phylum *Planctomycetes*, and four classes Bacteroidia, *Flavobacteria*,* SB‐1*, and *Sphingobacteria* within phylum *Bacteroidetes* (Fig. 9). These bacterial groups may serve as the significant contributors for DOC production in the near‐bottom water. *Alphaproteobacteria*,* OM190*,* Flavobacteria*, and *Sphingobacteria* also had highly negative correlations (−0.628 to −0.84) with salinity. Although the class *Gammaproteobacteria* was negatively correlated with both grain size and sampling depth (Fig. 9), the abundances of the genera *Vibrio* and *Pseudoalteromonas* within *Gammaproteobacteria* increased as grain size and sampling depth increased, which were in agreement with their high abundances at station A8 (Fig. 10). The main parameters significantly influencing the *Deltaproteobacteria* were salinity (0.734) and DOC (−0.72) (Fig. 9). Sedimentary types were identified as the important factors influencing the several bacterial taxonomic groups. For examples, sand sediments harbored more sequences affiliated with genera *Blastopirellula*,* Planctomyces*, and *Rhodopirellua* within phylum *Planctomycetes*. In contrast, the genus *Desulfobulbus* favored clay–silt sediments (Fig. 10). These results confirmed that compositions of specific bacterial taxonomic groups responded to the variation in environment parameters, including DOC, water depth, salinity, sedimentary grain size, and types in the hypoxic zone near the Changjiang Estuary and in the ECS.

### Possible mechanism of DO depletion contributed by sedimentary microbial community near the Changjiang Estuary

**Figure 11:**
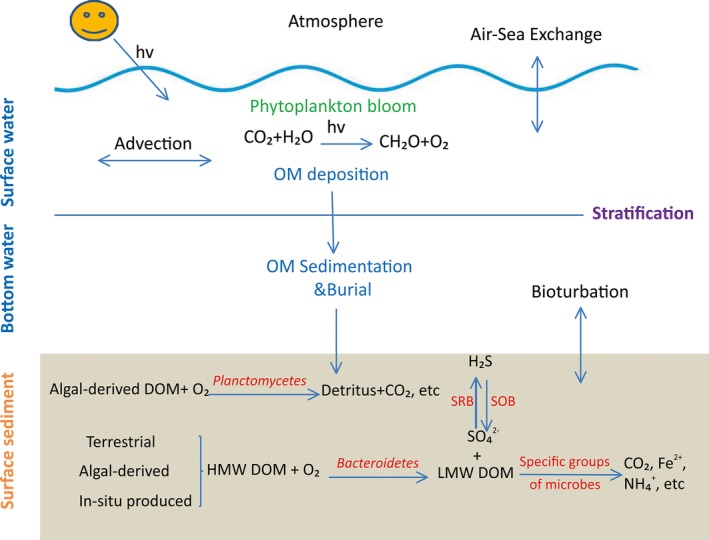
Possible mechanism of dissolved oxygen (DO) depletion contributed by the sedimentary microbial community in hypoxic zone near the Changjiang Estuary. The strong stratification prevents the vertical exchanges of organic matter and DO. Algal‐derived, terrestrial, and in situ‐produced organic matter in the sediments could be utilized by specific groups of microorganisms, such as aerobic *Planctomycetes* and *Bacteroidetes*. When the consumption exceeds DO supply, hypoxia develops.

Heterotrophs in the deep waters and in bottom sediments utilize a variety of pathways for the O_2_ consumption. On the basis of our results of geochemical and microbial molecular studies in the surface sediments of hypoxic zones near the mouth of Changjiang Estuary, we propose a possible mechanism of DO depletion contributed by sedimentary microbial community (Fig. 11). The strong stratification prevents the vertical exchanges of organic matter and DO. Algal‐derived, terrestrial, and in situ‐produced organic matter in the sediments could be utilized by specific groups of microorganisms, such as aerobic *Planctomycetes* and *Bacteroidetes*. When the consumption exceeds DO supply, hypoxia develops. We also found strong stratification at stations M7 and N6. However, phytoplankton blooms were not observed at these stations indicated a lower availability of algal‐derived organic matter. Thus, hypoxia failed to develop at these stations. Temperatures of the near‐bottom waters at stations M7 and N6 were relatively low, possibly inhibiting the respiration rate of microorganisms. For example, 9.73% sequences in station N6 had 98.9% similarity with the obligately aerobic *Lutibacter* sp. with optimum temperatures of ~25°C (Choi and Cho 2006). Thus, our results confirmed that the hypoxia near the Changjiang Estuary resulted from combination of physical, chemical, and biological processes.

## Conclusion

In this study, we detected newly hypoxic areas near the Changjiang Estuary during August 2013. To our knowledge, they are the first reports of the microbial community structures and diversity in the sediments near the Changjiang Estuary during hypoxia using the 16S rRNA‐based Illumina Miseq sequencing method. We have shown that sedimentary bacterial communities may have potential roles in the sulfur and carbon cycles at our studied sites. We found that decomposition of complex organic matter by specific groups of aerobic *Planctomycetes* and *Bacteroidetes* might contribute to O_2_ depletion in the strongly stratified areas. Hydrogen sulfide produced by SRB might be detoxified by putative *Gammaproteobacteria*‐affiliated SOB, which may serve as primary producers in the benthic food webs. In addition, the bacterial community structures were significantly impacted by the environmental variables, including sampling depth, DOC, sedimentary types, and salinity. Our results provided new insights into understanding further the mechanism of DO depletion contributed by the sedimentary microbial community near the Changjiang Estuary. Further efforts on the enrichment and isolation as well as metagenomics analysis will allow further insight into the ecological functions of the key microorganisms in the sediment underlying hypoxic conditions near the Changjiang Estuary and in the ECS.

## Conflict of Interest

None declared.

## Supporting information


**Figure S1.** Relative abundance (%) of the Classes within Phylum *Planctomycetes* in the surface sediment samples.

**Figure S2.** Relative abundance (%) of the Classes within Phylum *Bacteroidetes* in the surface sediment samples.

**Table S1.** Relative abundance (%) of the bacteria phyla or classes in the surface sediment samples.
**Table S2.** The relationship of microbial community structure to individual environmental variables revealed by CCA.
