# Supplementary figures and images for: Bacterial diversity in the surface sediments of the hypoxic zone near the Changjiang Estuary and in the East China Sea
Source: Microbiologyopen. 2016 Jan 27;5(2):323–39. doi: 10.1002/mbo3.330 (PMC4831476; doi:10.1002/mbo3.330)

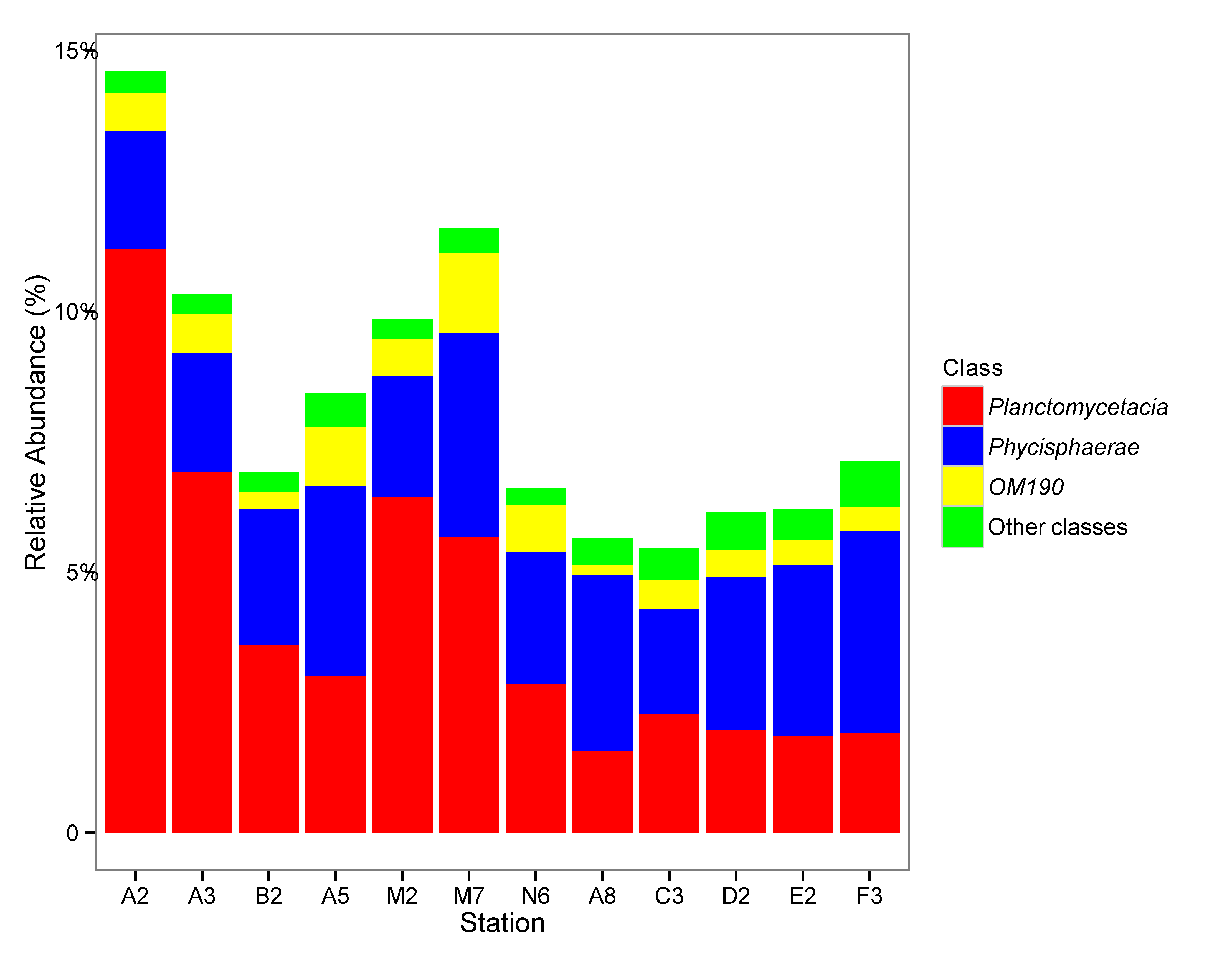

Supplement: Supplementary file 1 — Figure S1. Relative abundance (%) of the Classes within Phylum Planctomycetes in the surface sediment samples. [file MBO3-5-323-s001.tif]

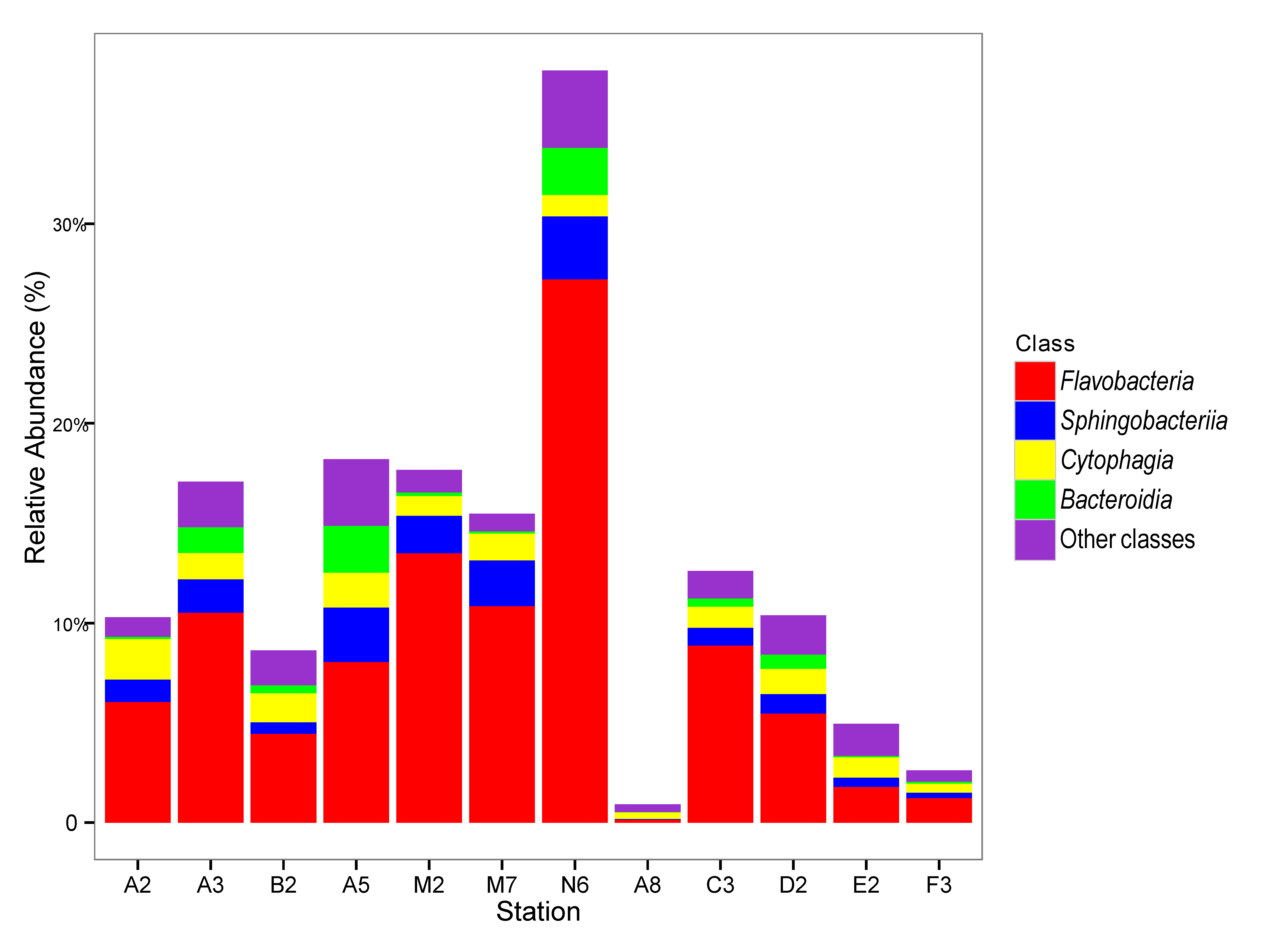

Supplement: Supplementary file 2 — Figure S2. Relative abundance (%) of the Classes within Phylum Bacteroidetes in the surface sediment samples. [file MBO3-5-323-s002.tif]
