# Supplementary material for: Bacterial diversity in the surface sediments of the hypoxic zone near the Changjiang Estuary and in the East China Sea
Source: Microbiologyopen. 2016 Jan 27;5(2):323–39. doi: 10.1002/mbo3.330 (PMC4831476; doi:10.1002/mbo3.330)
Supplement: Supplementary file 3 — Table S1. Relative abundance (%) of the bacteria phyla or classes in the surface sediment samples. Table S2. The relationship of microbial community structure to individual environmental variables revealed by CCA. [file MBO3-5-323-s003.docx]

Supplementary Table S1. Relative abundance (%) of the bacteria phyla or classes in the surface sediment samples.

|  | A2 | A3 | B2 | A5 | M2 | A8 | M7 | N6 | C3 | D2 | E2 | F3 |
| --- | --- | --- | --- | --- | --- | --- | --- | --- | --- | --- | --- | --- |
| *Acidobacteria* | 9.3 | 5 | 5.5 | 7.4 | 6.1 | 3.8 | 8.6 | 3.9 | 7 | 7.1 | 7.9 | 8.6 |
| *Actinobacteria* | 3.7 | 4.1 | 2 | 2 | 2.3 | 0.7 | 2.1 | 1.4 | 1.1 | 0.8 | 0.8 | 0.7 |
| *Bacteroidetes* | 10.3 | 17 | 8.6 | 18.2 | 17.6 | 0.9 | 15.4 | 37.6 | 12.6 | 10.4 | 5 | 2.6 |
| Candidate_division_OP8 | 0.5 | 0.2 | 1.4 | 0.4 | 0.2 | 9.2 | 0.2 | 0.1 | 0.2 | 0.4 | 0.4 | 1 |
| Candidate_division_WS3 | 2.7 | 2.4 | 2.2 | 3.1 | 2.4 | 1.4 | 2 | 1.2 | 2.6 | 3.1 | 2.6 | 2.2 |
| *Chlorobi* | 0.5 | 0.3 | 0.4 | 0.3 | 0.3 | 0.3 | 0.3 | 0.1 | 0.6 | 1.1 | 0.7 | 0.4 |
| *Chloroflexi* | 6.1 | 3.7 | 6.4 | 3 | 3.3 | 11.9 | 2.6 | 1.5 | 3.9 | 4.5 | 3.7 | 5.1 |
| *Cyanobacteria* | 0.7 | 1.3 | 0.7 | 0.9 | 1.3 | 0.1 | 0.5 | 0.5 | 1.9 | 1.4 | 0.2 | 0.1 |
| *Deferribacteres* | 0.4 | 0.3 | 0.6 | 0.7 | 0.3 | 1.3 | 0.4 | 0.1 | 0.6 | 1 | 0.7 | 1 |
| *Firmicutes* | 0.4 | 1.2 | 0.7 | 3.3 | 0.1 | 0.5 | 0.1 | 1.2 | 0.9 | 0.4 | 0.1 | 0.1 |
| *Gemmatimonadetes* | 1.2 | 0.9 | 1.1 | 1.6 | 1 | 0.9 | 2.2 | 1 | 1.8 | 1.7 | 2.5 | 1.8 |
| *Lentisphaerae* | 0.6 | 1.2 | 0.9 | 0.7 | 0.9 | 0.9 | 0.6 | 0.6 | 0.7 | 0.8 | 0.4 | 0.2 |
| *Nitrospirae* | 0.4 | 0.2 | 1.9 | 0.5 | 0.4 | 2.5 | 1 | 0.2 | 1.2 | 1.2 | 2.5 | 4.8 |
| *Planctomycetes* | 14.6 | 10.3 | 6.9 | 8.4 | 9.8 | 5.6 | 11.5 | 6.6 | 5.5 | 6.1 | 6.2 | 7.1 |
| *Alphaproteobacteria* | 4.8 | 4.6 | 2.5 | 4.3 | 5.6 | 3.4 | 6.4 | 4.7 | 3.5 | 2.8 | 3.7 | 3.8 |
| *Betaproteobacteria* | 0.2 | 0.1 | 0.1 | 0.3 | 0.1 | 0.1 | 1 | 0.4 | 0.3 | 0.4 | 0.3 | 0.3 |
| *Deltaproteobacteria* | 21.7 | 22 | 31.2 | 18.4 | 21.8 | 25 | 18.1 | 18.1 | 28.2 | 27.8 | 37.9 | 34 |
| *Epsilonproteobacteria* | 0.2 | 0.4 | 0.2 | 0.5 | 0.1 | 0.1 | 0.1 | 0.1 | 0.3 | 0.3 | 0.4 | 0.1 |
| *Gammaproteobacteria* | 18.5 | 21.4 | 15.3 | 22 | 23.3 | 12.7 | 24.1 | 17.9 | 24.2 | 22.8 | 20.8 | 19.3 |
| *Spirochaetae* | 0.3 | 0.5 | 1.3 | 1.1 | 0.4 | 2.5 | 0.1 | 0.9 | 0.3 | 0.6 | 0.6 | 0.7 |
| Unclassified | 1.7 | 1.5 | 8.7 | 1 | 1.9 | 14.4 | 1.1 | 0.9 | 1.6 | 4.3 | 1.5 | 5 |
| Other | 1.2 | 1.4 | 1.4 | 1.9 | 0.8 | 1.8 | 1.6 | 1 | 1 | 1 | 1.1 | 1.1 |

Supplementary Table S2. The relationship of microbial community structure to individual environmental variables revealed by CCA

|  | CCA1 | CCA2 | Pr^(1)^ | Significant code ^(2)^ |
| --- | --- | --- | --- | --- |
| DOC | -0.56423 | -0.8256 | 0.001 | *** |
| Depth | 0.9108 | -0.4130 | 0.006 | ** |
| Salinity | 0.6907 | 0.7231 | 0.024 | * |
| Clay/Silt | 0.4879 | -0.8729 | 0.039 | * |
| Mean Grain Size | 0.8582 | --0.5133 | 0.079 | - |
| Temperature | -0.-0.28857 | 0.9575 | 0.188 |  |
| DO | 0.5299 | -0.8481 | 0.267 |  |

The first two columns CCA1 and CCA2 give direction cosines of the vectors, showing the relationship between environmental variables and the ordination axes. (1). Pr shows the significance of correlation test of individual environmental variable and microbial communities; (2). Significant codes: ‘***’: 0 to 0.001; ‘**’: 0.001 to 0.01; ‘*’: 0.01 to 0.05; ‘-’: 0.05 to 1; The significance of the other categories was >0.1.
